# Supplementary material for: Deciphering cell cycle organization of Toxoplasma endodyogeny
Source: mBio. 2025 Jul 1;16(8):e01119-25. doi: 10.1128/mbio.01119-25 (PMC12345243; doi:10.1128/mbio.01119-25)
Supplement: Supplemental figures — Fig. S1 to S4. [file mbio.01119-25-s0001.pdf]

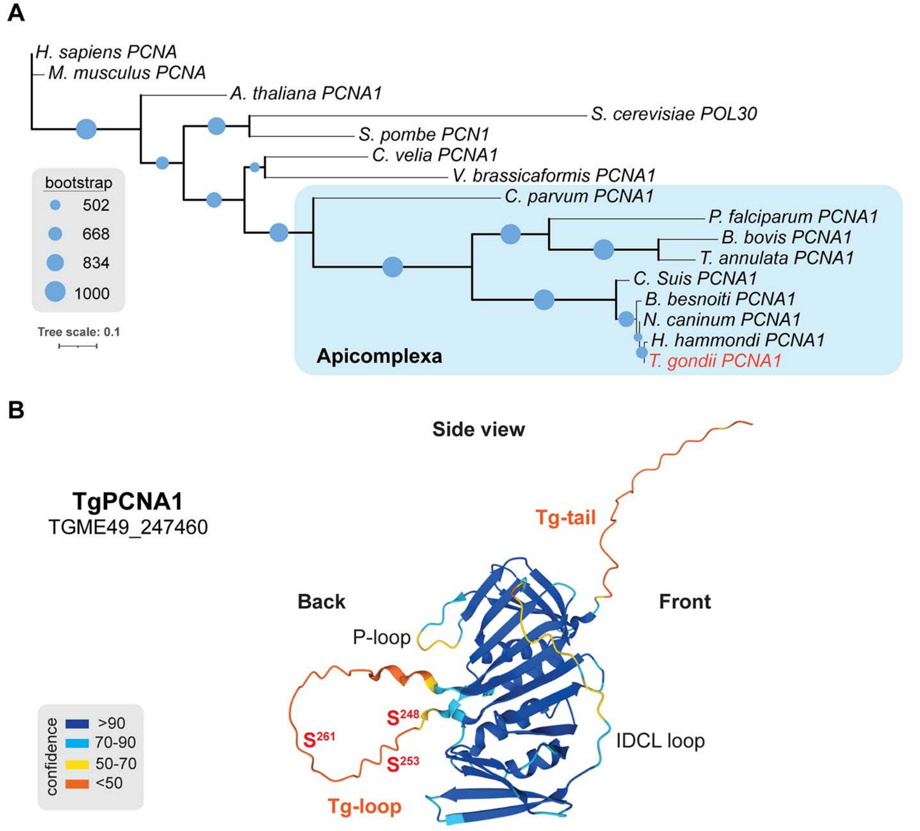

Figure S1. Toxoplasma PCNA1 is structurally and evolutionary conserved factor.
(A) Phylogenetic tree of PCNA1-related proteins.
(B) Folding prediction of TgPCNA1 (AlphaFold2). The structural deviations and predicted phosphorylation sites (ToxoDB.org) are shown.

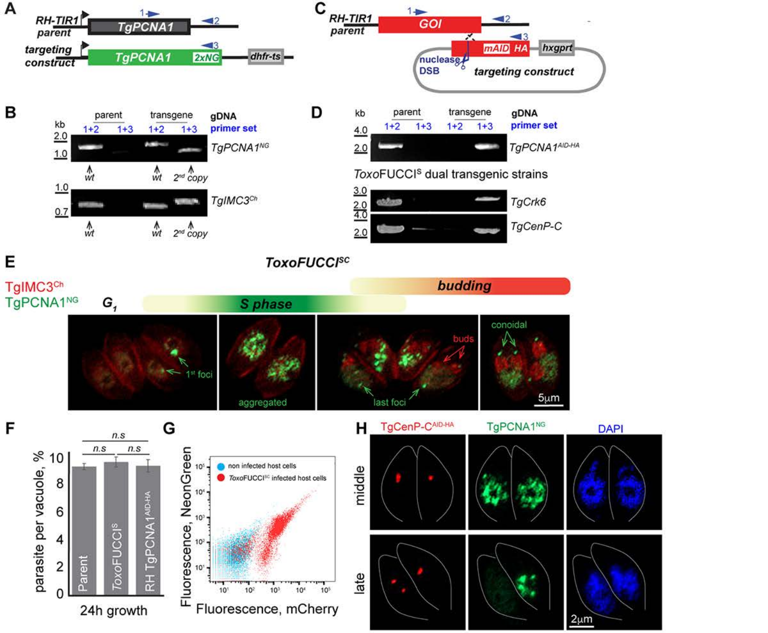

Figure S2. Construction of the ToxoFUCCI transgenic lines.
(A) Schematics for constructing ToxoFUCCI^{S} parasites. NeonGreen-tagged TgPCNA1 was introduced as a second copy under the control of the endogenous promoter. Schematics also indicate the relative positions of primers used to confirm expression of both TgPCNA1 copies (panel B).
(B and D). PCR analysis of the parental and indicated transgenic lines. Panel B: ToxoFUCCI^{S} and ToxoFUCCI^{SC}. Panel D: RHΔKu80TIR1 + TgPCNA1$^{AID\text{-}HA}$, ToxoFUCCI^{S} + TgCrk6$^{AID\text{-}HA}$, ToxoFUCCI^{S} + TgCenP-C$^{AID\text{-}HA}$. Primer combinations used to detect either native or recombined loci are shown.
(C) Schematics for constructing TgCrk4 and TgCenP-C AID-modified genes. Targeting plasmid included a 3' fragment of GOI genomic locus fused with encoded sequence for mini-version of AID (mAID), 3xHA (HA) epitopes, and the drug-selection marker hxgprt gene (grey box). Plasmid linearization with a unique endonuclease induced recombination at the GOI locus. Schematics also indicate the relative positions of the primers used to confirm GOI knock-in (panel D).
(E) Live fluorescence microscopy analysis of ToxoFUCCI^{SC} tachyzoites. The top schematic shows temporal expression of ToxoFUCCI markers.
(F) The replication rates of parental and transgenic lines. The average number of parasites per vacuole after 24 hours' growth was quantified in three independent experiments. Mean value -/+ SD are plotted on the graph.
(G) Flow cytometry analysis of uninfected and ToxoFUCCI^{SC}-infected HFF monolayers. The plot shows a fraction of fragmented host cells. Data was analyzed using FlowJo and the results of one of three independent experiments are shown.
(H) Individual images of the markers of ToxoFUCCIS tachyzoites expressing TgCenP-C$^{AID\text{-}HA}$ shown in Figure 3F (middle and late replication stages).

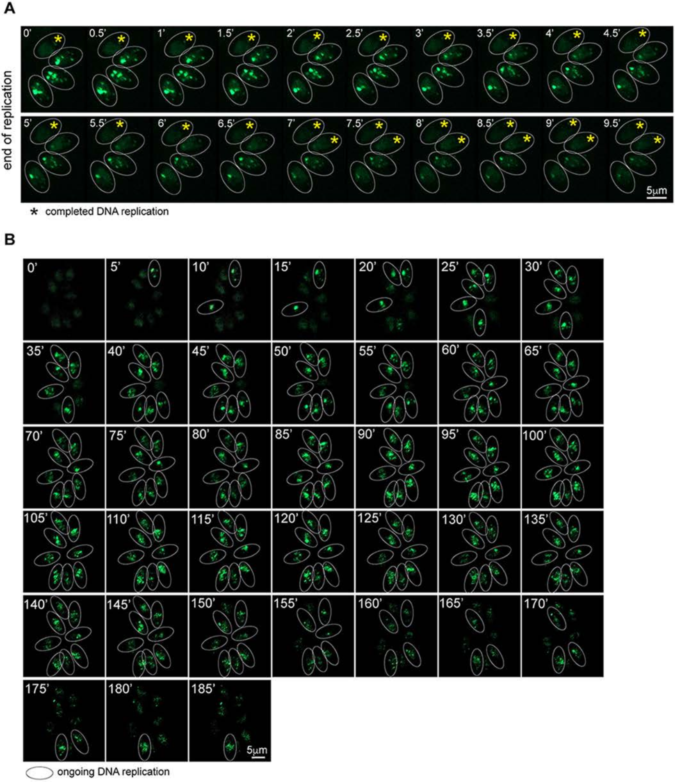

Figure S3. The dynamics of TgPCNA1^{NG} during tachyzoite replication.
(A) The end of DNA replication. The series of images depicts the offset of DNA replication in a vacuole of 4 parasites (last focus). Basal position of the nucleus and conoidal accumulation of ToxoFUCCI^{S} probe confirm that parasites are ending DNA replication. Note that individual parasites complete DNA replication at different times.
(B) The 3-hour progression of DNA replication. A series of images taken every 5 minutes shows the onset, progression, and ending of DNA replication in a vacuole of 8 parasites (last focus). Note that individual parasites start and complete DNA replication at different times.

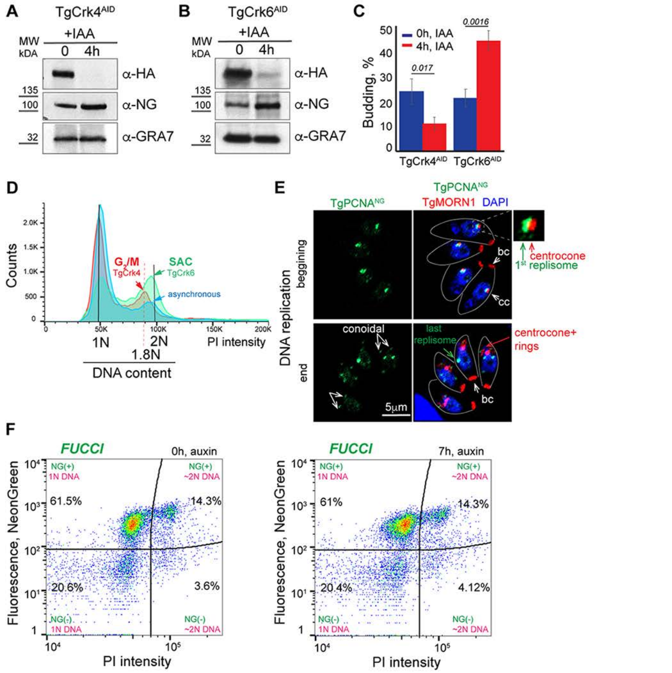

Figure S4. Validation of ToxoFUCCI lines.

(A and B) Western Blot analyses of the total lysates of the ToxoFUCCI^{S} tachyzoites expressing TgCrk4$^{AID\text{-}HA}$ (A) or TgCrk6$^{AID\text{-}HA}$ (B). Lysates of untreated parasites and parasites treated with 500 μM IAA for 4 hours were analyzed. Western blots were probed with α-HA and α-NeonGreen, and with α-GRA7 to confirm equal loading of the total lysates.

(C) Quantification of the budding populations of ToxoFUCCI^{S} + TgCrk4$^{AID\text{-}HA}$ and ToxoFUCCI^{S} + TgCrk6$^{AID\text{-}HA}$ tachyzoites during cell cycle block (4 hours, IAA). 100 random vacuoles of parasites were examined for α-TgIMC1-positive internal buds in three independent experiments. Mean -/+ SD values of three independent experiments are plotted on the graph.

(D) FACScan analysis of DNA content of parasites asynchronously growing (blue), and arrested at G_2/M (red: ToxoFUCCI^{S} + TgCrk4$^{AID\text{-}HA}$, 4 hours IAA) or SAC (green: ToxoFUCCI^{S} + TgCrk6$^{AID\text{-}HA}$, 4 hours IAA) checkpoints. Note the differences in DNA content of the checkpoint-arrested populations. The results of one of three independent experiments are shown.

(E) Immunofluorescent microscopy analysis of ToxoFUCCI^{S} tachyzoites. Images depict the beginning and end of DNA replication. Parasites were stained with α-TgMORN1. The markers' colocalization is shown in the enlarged image on the side. Note that ToxoFUCCI^{S} probe accumulates on the conoids of the daughter cells surrounded by the TgMORN1-positive rings.

(F) Flow cytometry analysis of ToxoFUCCI^{S} tachyzoites grown at the indicated conditions. Plots show no changes in TgPCNA1^{NG} expression and DNA content (PI). Data was analyzed in FlowJo and the results of one of three independent experiments are shown.
