# Supplementary material for: Deciphering cell cycle organization of Toxoplasma endodyogeny
Source: mBio. 2025 Jul 1;16(8):e01119-25. doi: 10.1128/mbio.01119-25 (PMC12345243; doi:10.1128/mbio.01119-25)
Supplement: Captions — for supplemental tables and movies. [file mbio.01119-25-s0002.docx]

**Table S1.** Primers, transgenic strains used in the study, and PCNA1 orthologs.

**Table S2.** TgPCNA1 proteomes.

**Table S3.** Raw data used in the Figures and Supplemental Figures.

**Table S4.** *Toxo*FUCCI real-time microscopy analysis.

**Movie S1.** Beginning of DNA replication.

**Movie S2.** End of DNA replication.
